# Highly sensitive magnetic particle imaging of vulnerable atherosclerotic plaque with active myeloperoxidase-targeted nanoparticles

**DOI:** 10.7150/thno.49812

**Published:** 2021-01-01

**Authors:** Wei Tong, Hui Hui, Wenting Shang, Yingqian Zhang, Feng Tian, Qiang Ma, Xin Yang, Jie Tian, Yundai Chen

**Affiliations:** 1 Medical School of Chinese PLA, Chinese PLA General Hospital, Beijing, 100853, China.; 2 Department of Cardiology, the Sixth Medical Centre, Chinese PLA General Hospital, Beijing, 100853, China.; 3 CAS Key Laboratory of Molecular Imaging, Institute of Automation, Chinese Academy of Sciences, Beijing, 100190, China.; 4 University of Chinese Academy of Sciences, Beijing, China.; 5 Beijing Advanced Innovation Center for Big Data-Based Precision Medicine, School of Medicine, Beihang University, Beijing, 100083, China.

**Keywords:** myeloperoxidase, vulnerable plaque, magnetic particle imaging, fluorescence imaging, computed tomographic angiography

## Abstract

Inflammation is a pivotal driver of atherosclerotic plaque progression and rupture and is a target for identifying vulnerable plaques. However, challenges arise with the current *in vivo* imaging modalities for differentiating vulnerable atherosclerotic plaques from stable plaques due to their low specificity and sensitivity. Herein, we aimed to develop a novel multimodal imaging platform that specifically targets and identifies high-risk plaques *in vivo* by detecting active myeloperoxidase (MPO), a potential inflammatory marker of vulnerable atherosclerotic plaque.

**Methods:** A novel multimodal imaging agent, 5-HT-Fe_3_O_4_-Cy7 nanoparticles (5HFeC NPs), used for active MPO targeting, was designed by conjugating superparamagnetic iron oxide nanoparticles (SPIONs) with 5-hydroxytryptamine and cyanine 7 N-hydroxysuccinimide ester. The specificity and sensitivity of 5HFeC NPs were evaluated using magnetic particle imaging (MPI), fluorescence imaging (FLI), and computed tomographic angiography (CTA) in an ApoE^-/-^ atherosclerosis mouse model. Treatment with 4-ABAH, an MPO inhibitor, was used to assess the monitoring ability of 5HFeC NPs.

**Results:** 5HFeC NPs can sensitively differentiate and accurately localize vulnerable atherosclerotic plaques in ApoE^-/-^ mice *via* MPI/FLI/CTA. High MPI and FLI signals were observed in atherosclerotic plaques within the abdominal aorta, which were histologically confirmed by multiple high-risk features of macrophage infiltration, neovascularization, and microcalcification. Inhibition of active MPO reduced accumulation of 5HFeC NPs in the abdominal aorta. Accumulation of 5HFeC NPs in plaques enabled quantitative evaluation of the severity of inflammation and monitoring of MPO activity.

**Conclusions:** This multimodal MPI approach revealed that active MPO-targeted nanoparticles might serve as a method for detecting vulnerable atherosclerotic plaques and monitoring MPO activity.

## Introduction

Atherosclerosis is a systematic, progressive, inflammatory disease that causes significant morbidity and mortality worldwide 1. Complications related to atherosclerosis, such as ischemic stroke and myocardial infarction (MI), the latter of which is the leading cause of death in developed countries, are primarily initiated by inflammation-driven rupture of vulnerable atherosclerotic plaques 2. Imaging techniques, particularly *in vivo* molecular imaging techniques, play an important role in risk stratification of atherosclerotic plaques 3.

Currently, advanced molecular imaging techniques, such as ultrasound, positron emission tomography (PET), and magnetic resonance imaging (MRI), are being used for imaging vulnerable plaques, particularly since the development of novel imaging probes targeting inflammation and microcalcification 4-6. However, each imaging modality has limitations. Ultrasound has the advantage of low cost and real-time imaging; however, it also suffers from limited tissue penetration 7. Meanwhile, MRI techniques are limited by motion effects associated with the cardiac cycle and diaphragmatic movements when utilized for coronary plaque imaging 8. Although PET offers high sensitivity, it is impaired by radiation exposure and low spatial resolution 9, 10. Furthermore, both MRI and PET require a long acquisition time. An ideal atherosclerosis imaging platform uses high sensitivity imaging techniques and a specific targeting marker to identify vulnerable plaques 11. Therefore, multiple imaging modalities integrated with multimodal imaging tracers are urgently needed to identify high-risk plaques.

Magnetic particle imaging (MPI) is an emerging tomographic method that uses superparamagnetic iron oxide nanoparticles (SPIONs) as imaging agents 12-14. Since its first introduction in 2005 15, high mass sensitivity (Fe nanograms), high spatial resolution (sub-mm), zero tissue depth signal attenuation, and lack of ionizing radiation have been reported as advantages of MPI 16. Furthermore, iron oxide nanoparticles have been approved for clinical applications 17-19. Computed tomographic angiography (CTA) can provide anatomical information for the vessel and directly assess luminal stenosis severity. These advantages make MPI/CTA a promising imaging modality to identify vulnerable plaques in arteries. Furthermore, *in vivo* fluorescence imaging (FLI) has distinct advantages, such as high sensitivity, no ionization, and relatively simple operation 11. Thus, FLI/MPI/CTA may serve as a promising multimodality technique for accurately identifying vulnerable plaques with high sensitivity and spatial resolution.

Myeloperoxidase (MPO), an inflammatory protein, is secreted as an active enzyme by neutrophils and macrophages during inflammation in atherosclerotic plaques 20, 21. Plasma MPO protein is positively associated with the presence and severity of coronary artery disease 22, 23 as well as with patient prognosis 24. In fact, an autopsy study revealed that MPO is abundantly expressed in ruptured coronary plaques among patients who died of acute MI 25. Moreover, advanced human atheromas exhibit higher levels of MPO-expressing macrophages than early-stage atherosclerotic lesions 21. Recent animal studies have reported that inhibition of MPO activity can stabilize vulnerable plaques by reducing intraplaque hemorrhage and increasing fibrous cap thickness 26. Therefore, MPO is increasingly becoming recognized as a potential target for the identification of vulnerable atherosclerotic plaques. Moreover, imaging agents composed of 5-hydroxytryptamine (5-HT) can reportedly self-oligomerize or bind proteins when catalyzed by active MPO in inflamed tissues 27, suggesting that it has potential for application as an imaging biomarker of vulnerable plaques.

In this study, we aimed to develop a novel multimodal atherosclerotic plaque-imaging platform that specifically targets and identifies high-risk plaques *in vivo* by detecting the distribution of active MPO during inflammation in atherosclerotic plaques (Figure S1). A novel multimodal imaging agent, 5-HT- Fe_3_O_4_-Cy7 nanoparticles (5HFeC NPs), used for active MPO targeting, was designed by conjugating 5-HT and Fe_3_O_4_@PEG-COOH with cyanine 7 N-hydroxysuccinimide ester (Cy7-NHS). The *in vivo*-specific targeting ability of 5HFeC NPs was evaluated in an established MPO-implanted mouse model using FLI and MPI. FLI and MPI/CTA imaging of active MPO in atherosclerotic plaques was performed in the atherosclerotic ApoE^-/-^ mouse model, in which 5HFeC NPs showed high contrast enhancement in plaques of the abdominal aorta with histologically confirmed high-risk features of macrophage infiltration, neovascularization, and microcalcification. FLI and MPI were further used to confirm the targeting ability of 5HFeC NPs after inhibition of MPO activity in atherosclerotic ApoE^-/-^ mice.

## Results

### Characterization of 5HFeC NPs

**Figure 1:**
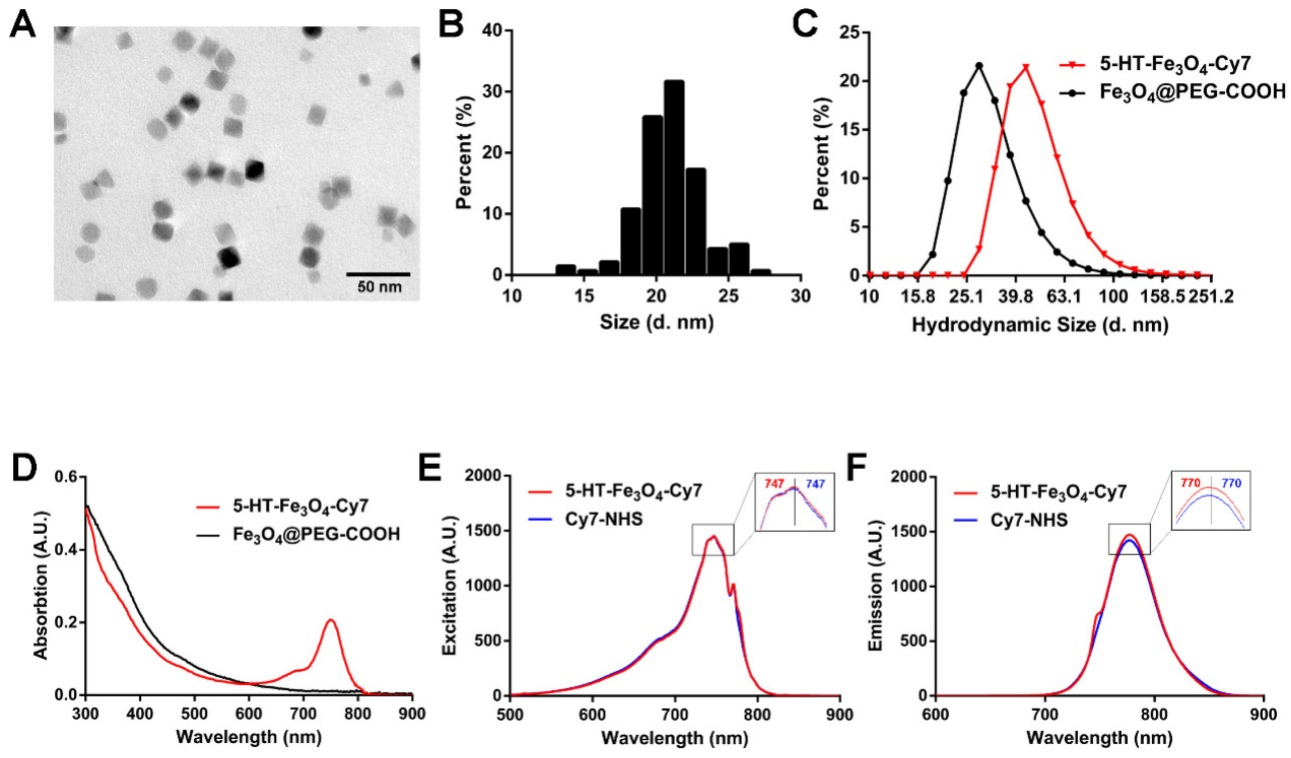
** Characterization of 5HFeC NPs.** TEM image (A) and size distribution (B) of 5HFeC NPs. Hydrodynamic sizes (C) and UV-VIS-NIR spectra (D) of 5HFeC NPs and Fe_3_O_4_@PEG-COOH. Excitation spectra (E) and emission spectra (F) of 5HFeC NPs and Cy7-NHS.

**Figure 2:**
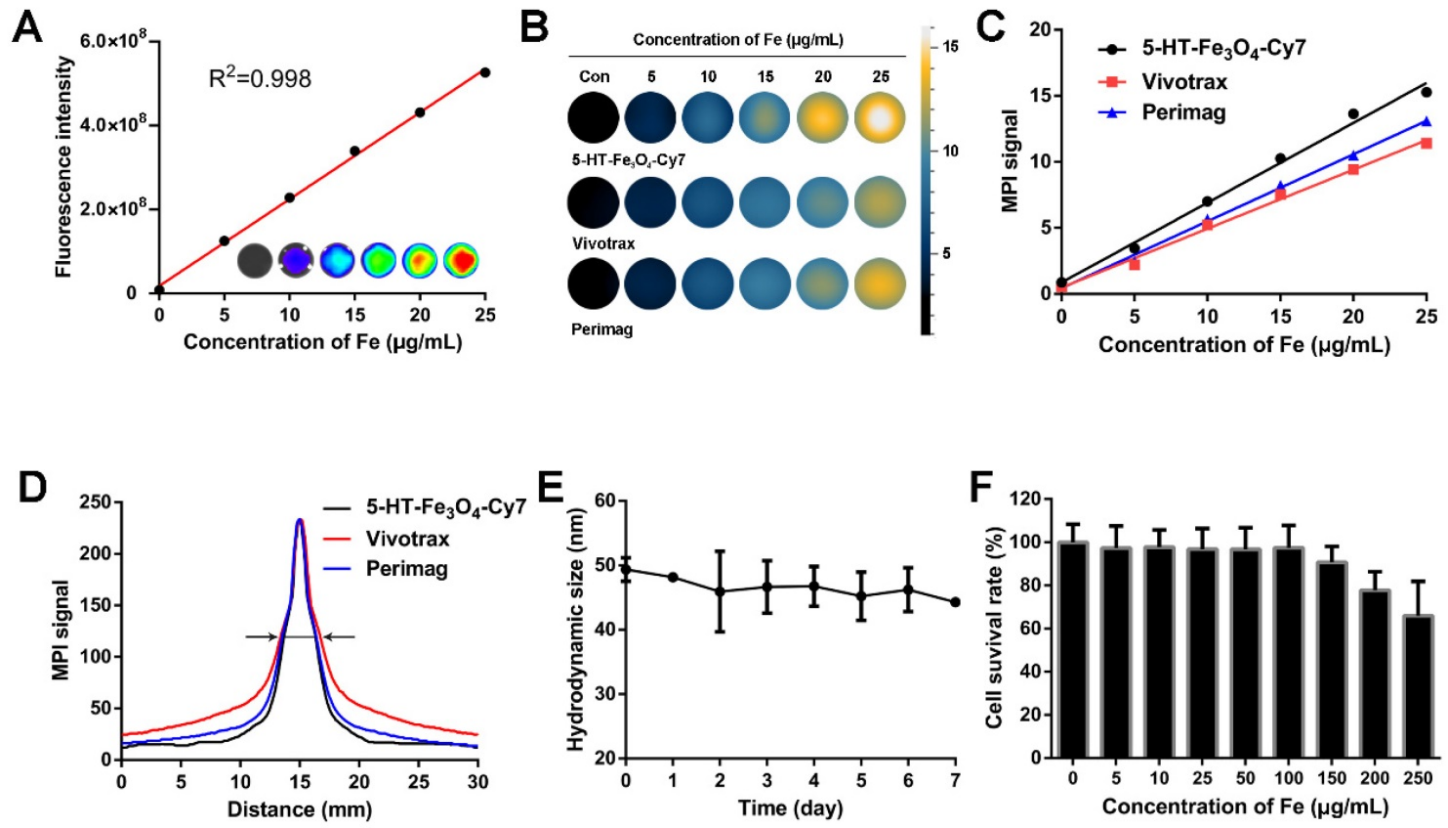
** 5HFeC NPs characterization, stability, and toxicity tests.** Standard curve generated by quantifying fluorescence intensity of various concentrations of 5HFeC NPs (A). MPI images of 5HFeC NPs, Vivotrax, and Perimag at various concentrations (B). Standard curve generated by quantifying MPI signal of various concentrations of 5HFeC NPs, Vivotrax, and Perimag (C). Point spread function curve of 5HFeC NPs, Vivotrax, and Perimag (D). Spatial resolutions of the 5HFeC NPs, Vivotrax, and Perimag were analytically estimated as the full width at half-maximum 2.76 mm, 3.45 mm, and 2.97 mm, respectively. Hydrodynamic size of 5HFeC NPs at room temperature over 7 days (E). *In vitro* cytotoxicity of RAW 264.7 cells incubated with various concentrations of 5HFeC NPs (F).

To synthesize an active MPO-specific imaging probe, 5HFeC NPs were first prepared and characterized. A transmission electron microscopy (TEM) image of 5HFeC NPs is shown in Figure 1A; the nanoparticles were mono-dispersed and had an average size of 20.97 ± 2.23 nm in the dry state (Figure 1B). The powder X-ray diffraction (XRD) patterns in Figure S2A reveal that 5HFeC NPs are cubic nanocrystals, with the space group of Fd-3m. Figure 1C shows that the average hydrodynamic size of Fe_3_O_4_@PEG-COOH was 32.94 ± 12.65 nm, whereas that of 5HFeC NPs was 50.27 ± 18.96 nm. Moreover, Fe_3_O_4_@PEG-COOH had a negative zeta potential of -24.5 ± 8.5 mV. After coating with 5-HT and Cy7-NHS, the zeta potential of 5HFeC NPs was reduced to -31.8 ± 8.9 mV. Fourier transform infrared (FT-IR) spectroscopy was performed to ensure the conjugation of Fe_3_O_4_@PEG-COOH with 5-HT and Cy7-NHS. The absorption peak of 5-HT-Fe_3_O_4_ at 1737 cm^-1^ was lower than that of Fe_3_O_4_ @PEG-COOH (Figure S2B), which is the characteristic peak of C=O (COOH). However, at 1654 cm^-1^ (the characteristic peak of C=O (CONH)), the absorption peak of 5-HT-Fe_3_O_4_ NPs was higher than that of Fe_3_O_4_ @PEG-COOH. These results indicate that 5-HT was successfully conjugated with Fe_3_O_4_ @PEG-COOH. When 5-HT-Fe_3_O_4_ NPs and Cy7-NHS were further connected, no noticeable stretching vibration peaks were observed in the corresponding bands since the bound agents occupy the amino group. Optical absorption spectra analysis showed that the 5HFeC NPs had an absorption peak at 749 nm (Figure 1D). To select a suitable fluorescent filter, the excitation (747 nm) and emission (770 nm) spectra of 5HFeC NPs were analyzed and were consistent with the spectra of free Cy7 (Figure 1E and 1F). Fluorescence intensity and MPI signal at different concentrations of 5HFeC NPs were recorded (Figure 2A-2C). In the range of 0-25 μg/mL, both the fluorescence intensity (Y = 2.07 × 10^7^ X + 1.79 × 10^7^, R^2^ = 0.998, P < 0.001) and MPI signal (Y = 0.60X + 0.87, R^2^ = 0.992, P < 0.001) of 5HFeC NPs showed a linear correlation with the concentration of Fe. Moreover, MPI signals of Vivotrax (Magnetic Insight Inc., USA) and Perimag (Micromod Partikeltechnologie GmbH, Germany) at concentrations equal to that of Fe were lower than the MPI signal of 5HFeC NPs (Figure 2B and 2C). Figure 2D shows the point spread function (PSF) curves of 5HFeC NPs, Vivotrax, and Perimag; the spatial resolutions of 5HFeC NPs, Vivotrax, and Perimag were 2.76, 3.45, and 2.97 mm, respectively.

### *In vitro* stability, biocompatibility, and specificity of 5HFeC NPs

**Figure 3:**
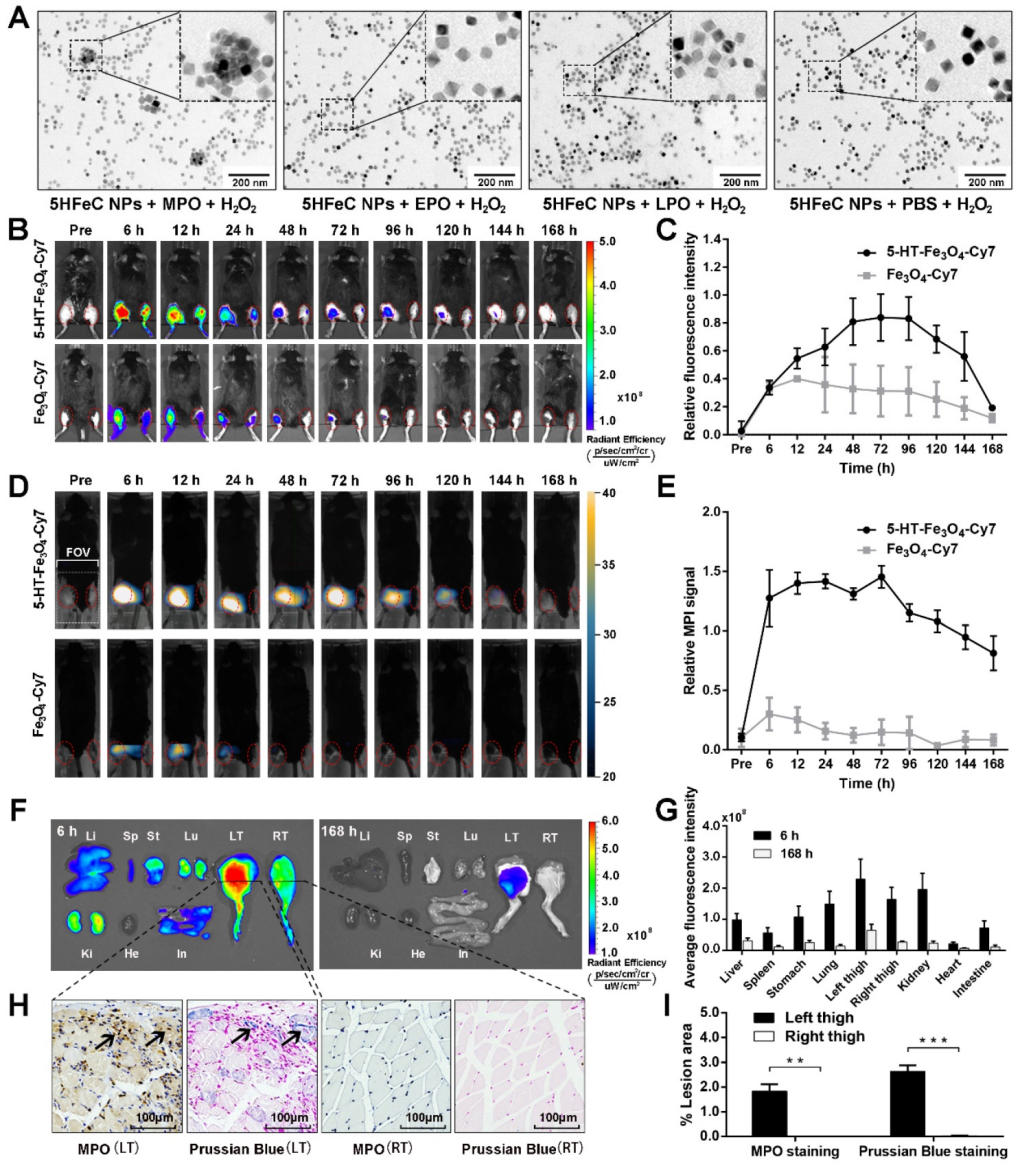
***In vitro and in vivo* MPO-targeting specificity of 5HFeC NPs.** TEM images of 5HFeC NPs after incubation with H_2_O_2_ and MPO, EPO, LPO, or PBS (A). *In vivo* FLI over 168 h of MPO-implanted mice intravenously administered 5HFeC NPs or Fe_3_O_4_-Cy7 (B). Red ellipse refers to the region of interest (ROI) in thighs. Relative fluorescence intensity of left thigh to right thigh (C). MPI imaging over 168 h of mice intravenously administered 5HFeC NPs or Fe_3_O_4_-Cy7 (D). MPI field of view (6 × 4 cm) is noted in the figure. Red ellipse denotes ROI in the thighs. Relative MPI signal of left thigh to right thigh (E). *Ex vivo* imaging of major organs at 6 and 168 h post-injection (F). Li, liver; Sp, spleen; St, stomach; Lu, lung; LT, left thigh; RT, right thigh; Ki, kidney; He, heart; In, intestine. Average fluorescence intensity of different tissues (G). MPO immunohistochemical and Prussian blue staining of muscle tissues from mouse thighs implanted with MPO plus glucose oxidase (left) or PBS (right) at 6 h post-injection of 5HFeC NPs (H). Quantification analysis of MPO immunohistochemical and Prussian blue staining (I). (n = 3 for the MPO-implanted mice; **: P < 0.005; ***: P < 0.001).

To determine the stability of 5HFeC NPs, the nanoparticle solution was dissolved in phosphate-buffered saline (PBS) for one week at room temperature (20 ℃). The hydrodynamic size of the nanoparticles did not exhibit significant differences over one week, demonstrating that their sizes remained stable in PBS (Figure 2E). The cell counting kit-8 (CCK-8) assay was performed to examine the toxic effects of 5HFeC NPs on RAW 264.7 cells. The viability of RAW 264.7 cells incubated with 5HFeC NPs (0-250 μg/mL) is shown in Figure 2F. No noticeable cytotoxicity (< 100 μg/mL) was observed in these cells. Furthermore, *in vitro* assessment of specificity against oxidases, including MPO, EPO, LPO, and PBS, as a control, was performed (Figure 3A). Following incubation of 5HFeC NPs with MPO and H_2_O_2_ for 1 h at 37 °C, TEM analysis revealed that 5HFeC NPs clustered together to form larger nanoparticles. However, when incubated with H_2_O_2_, and EPO, LPO or PBS, 5HFeC NPs did not aggregate into larger nanoparticles.

### FLI/MPI/CT imaging of MPO-implanted mice to confirm the specificity and biodistribution of 5HFeC NPs* in vivo* and* ex vivo*

To determine the specificity of 5HFeC NPs to target active MPO *in vivo*, MPO and glucose oxidase were embedded in the left thigh of C57BL/6J mice, while the right thigh only received PBS. For the MPO-implanted mice injected with 5HFeC NPs (Figure 3B), fluorescence intensity continuously increased in the left thigh (implanted with MPO) compared to that in the right thigh from 6 to 168 h after nanoparticle injection. The amount of 5HFeC NPs accumulated in the left thigh began increasing 6 h post-injection and gradually reached a peak (0.84-fold increase compared to the right thigh) at 72 h post-injection (Figure 3C). Similarly, the injection of Fe_3_O_4_-Cy7 also resulted in an increase in fluorescence intensity in the left thigh compared with that in the right thigh from 6 to 168 h (Figure 3B). However, the relative fluorescence intensity in the mice injected with Fe_3_O_4_-Cy7 was lower than that in mice injected with 5HFeC NPs, with a maximum relative fluorescence intensity of 0.40 at 12 h post-injection in mice injected with Fe_3_O_4_-Cy7 (Figure 3C). MPI signal was continuously detected in the left thigh from 6 to 168 h post-injection of 5HFeC NPs (Figure 3D). At 6 h post-injection, the MPI signal was clearly detected in the left thigh (Figure 3E) and a 1.45-fold increase was observed at 72 h post-injection, after which point the signal began to decrease. Therefore, 6 h to 72 h post-injection was the optimal window to visualize active MPO *via* MPI imaging. Additionally, three-dimensional (3D) co-registration of the MPI and CT images enabled the visualization of 5HFeC NP distribution in the MPO-implanted thigh (Supplementary movie 1). For the mice injected with Fe_3_O_4_-Cy7, MPI signal was detected in the left thigh only at 6, 12 and 24 h post-injection (Figure 3D). The relative MPI signal was also lower at the same time points in mice injected with 5HFeC NPs. A 0.30-fold increase in the MPI signal was recorded at 6 h post-injection, after which point the signal began to decrease (Figure 3E).

The distribution of 5HFeC NPs was further examined in harvested tissues at 6 and 168 h post-injection (Figure 3F). The left thigh showed the highest fluorescence intensity among all tissues both at 6 and 168 h, confirming the specificity of 5HFeC NPs for MPO (Figure 3G). As shown in Figure 3H and 3I, immunohistochemical staining of MPO revealed abundant MPO in the left thigh, but not in the right thigh (P < 0.005). Prussian blue staining further confirmed that more 5HFeC NPs accumulated in the muscle tissues of the left thigh than in those of the right thigh (P < 0.001). Moreover, fluorescence was detected in the kidney, lung, stomach, liver, and spleen at 6 h post-injection. Prussian blue staining of the main organs at 6 h post-injection indicated that 5HFeC NPs were primarily deposited in the liver and spleen, and not in the kidney, lung, or heart (Figure S3).

### FLI/MPI/CTA imaging of active MPO to identify aortic plaque in atherosclerotic ApoE^-/-^ mice

**Figure 4:**
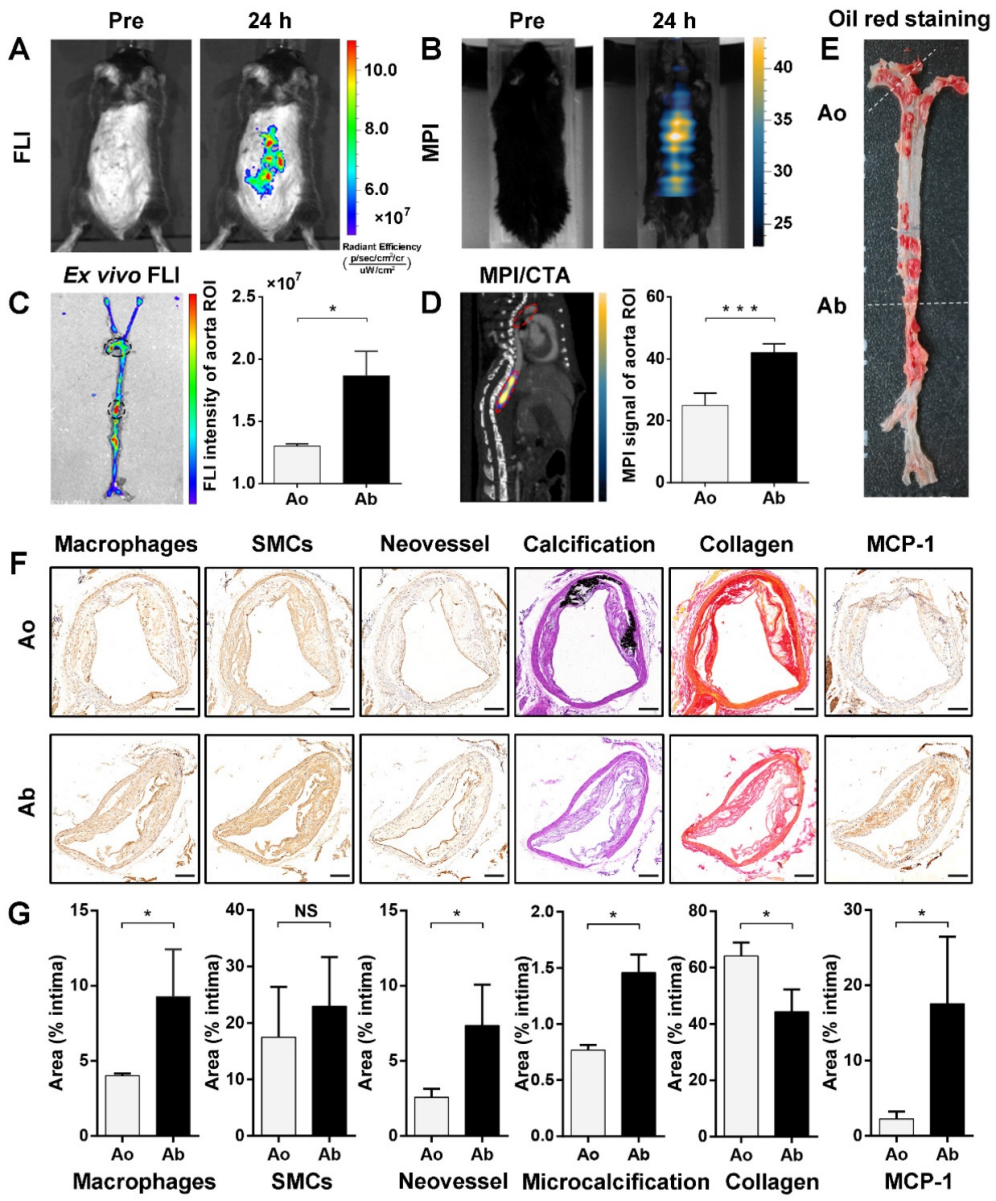
** Identification of vulnerable plaque located in abdominal aorta of atherosclerotic mice *via* FLI/MPI/CTA imaging after intravenous injection of 5HFeC NPs.** *In vivo* FLI of active MPO in aorta atheroma of ApoE^-/-^ mice before, and 24 h post, injection of 5HFeC NPs (A). MPI imaging of active MPO in aorta atheroma of ApoE^-/-^ mice before and 24 h post-injection of 5HFeC NPs (B). *Ex vivo* FLI of active MPO in the aorta of atherosclerotic mice and quantification of fluorescence intensity in aortic arch and abdominal aorta (C). Black ellipse indicates ROI in the aorta. MPI/CTA imaging of active MPO in the aorta of atherosclerotic mice and quantification of MPI signal in aortic arch and abdominal aorta (D). Red ellipse represents ROI in the mouse. Oil Red O staining images of the aorta from atherosclerotic mice (E). Cross-sections of plaque segments collected from aortic arch and abdominal aorta of atherosclerotic mouse and stained for macrophages (CD68), SMCs (ɑ-SMA), neovessel (CD31), calcification (von Kossa), collagen (picrosirius red), and MCP-1. SMCs, smooth muscle cells; MCP-1, monocyte chemoattractant protein-1-positive cells (F). Quantification of macrophages, SMCs, neovessel, microcalcification, collagen, and MCP-1 in the intimal area of plaque segments collected from aortic arch and abdominal aorta (G). Ao, aortic arch; Ab, abdominal aorta; scale bar in (F), 200 μm. (n = 3 for the atherosclerotic mice; NS: non-significant differences; *: P < 0.05; ***: P < 0.001).

Eight-week-old male ApoE^-/-^ mice were used for imaging after 40‒42 weeks of a high-fat and high-cholesterol diet (HFD). Fluorescence images *in vivo* were acquired before, and at 24 h after, intravenous injection of 5HFeC NPs (Figure 4A). MPI images were also acquired before, and at 24 h post-injection (Time window of MPI imaging: 6 h to 72 h post-injection; Figure 4B). The biodistribution of 5HFeC NPs in harvested tissues from the atherosclerotic mice was analyzed at 24 h post-injection (Figure S4A). Aside from the kidney, the highest fluorescence intensity was detected in the aorta (Figure S4B). Further *ex vivo* fluorescence images of the aorta revealed fluorescent nanoparticle-derived intensity primarily at the abdominal aorta (Figure 4C). To image the 3D distribution of 5HFeC NPs in the aorta of mice, whole-body 3D MPI was performed and co-registered with CTA (Figure S5 and Supplementary movie 2). MPI/CTA images showed that an Fe_3_O_4_-derived MPI signal was mainly detected at the abdominal aorta (Figure 4D). Quantitative analysis of the region of interest (ROI) revealed that both fluorescence intensity (1.87 ± 0.11 × 10^7^ *vs.* 1.30 ± 0.01 × 10^7^, P < 0.05, respectively) and the MPI signal (42.04 ± 1.26 *vs.* 24.95 ± 2.27, P < 0.001, respectively) were higher in the abdominal aorta than in the aortic arch.

### Component and vulnerability analysis of plaques in the aorta

Oil O Red staining of the aorta showed that the atherosclerotic plaque was primarily observed in both the aortic arch and abdominal aorta (Figure 4E). The immunohistochemical, von Kossa and picrosirius red staining were performed to investigate the plaque components and vulnerability of the aortic arch and abdominal aorta, including macrophages, smooth muscle cells, neovessels, microcalcification, and MCP-1. As shown in Figure 4F and 4G, lesions in the abdominal aorta contained more macrophages (9.28 ± 3.15% *vs.* 4.02 ± 0.14%, P < 0.05, respectively) and neovessels (7.36 ± 2.71% *vs.* 2.58 ± 0.57%, P < 0.05, respectively), but had less collagen (44.37 ± 7.84% *vs.* 64.17 ± 4.71%, P < 0.05, respectively) than did those in the aortic arch. Meanwhile, smooth muscle cells showed no relative difference between lesions in the abdominal aorta and aortic arch (22.98 ± 8.69% *vs.* 17.48 ± 8.90%, P > 0.05, respectively). Moreover, the amount of calcification was significantly higher in aortic arch lesions than in those of the abdominal aorta (16.18 ± 0.63% *vs.* 1.80 ± 0.51%, P < 0.001, respectively). CTA images of atherosclerotic mice also showed calcification in the aortic arch (Figure S6A), but not in the abdominal aorta (Figure S6B). Arterial calcification has been shown to correlate with cardiovascular events 28. Moreover, larger calcifications (>200 μm) may contribute to plaque stabilization, whereas microcalcifications (1-30 μm) may destabilize atherosclerotic plaques 29, 30. Therefore, the distribution of large calcification and microcalcification was analyzed in calcified areas in the aortic arch and abdominal aorta. Despite the abundance of calcification in the aortic arch, large calcifications (> 200 μm) accounted for 95.25 ± 0.05% of the calcified area in lesions of the aortic arch. Quantitative analysis revealed that microcalcification (1-30 μm) had a smaller distribution in the aortic arch than in the abdominal aorta (0.77 ± 0.03% *vs.* 1.46 ± 0.16%, P < 0.05, respectively). The expression of monocyte chemoattractant protein-1 (MCP-1), a predicted mediator of human plaque vulnerability 31, was further examined to confirm the vulnerability of plaques. MCP-1 was significantly higher in lesions in the abdominal aorta than in those of the aortic arch (17.57 ± 8.86% *vs.* 2.27 ± 0.98%, P < 0.05, respectively).

### FLI/MPI imaging to monitor MPO activity in atherosclerotic plaques

Eight-week-old male ApoE^-/-^ mice were administered the specific irreversible MPO inhibitor 4-aminobenzoic acid hydrazide (4-ABAH; 40 mg/kg) by intraperitoneal injection twice per day in addition to being fed HFD (HFD + 4-ABAH group). Eight-week-old male C57BL/6J mice were fed the standard laboratory chow diet as a control group (Con group). After 40‒42 weeks, the carotid arteries and aorta were isolated and analyzed by Oil O Red staining (Figure S7A). The plaque area was significantly higher in the HFD group than in the Con group (ratio of plaque area to whole aorta area: 55.52 ± 5.80% in HFD group *vs.* 20.83 ± 1.73% in Con group, P < 0.001; Figure S7B). However, administration of the MPO inhibitor 4-ABAH resulted in no differences in the plaque area (HFD group: 55.52 ± 5.80% *vs.* HFD + 4-ABAH group: 48.51 ± 3.37%, P > 0.05). Hematoxylin and eosin (H&E) staining of the abdominal aorta confirmed similar thickness of plaques between the HFD group and HFD + 4-ABAH group (Figure S7C).

**Figure 5:**
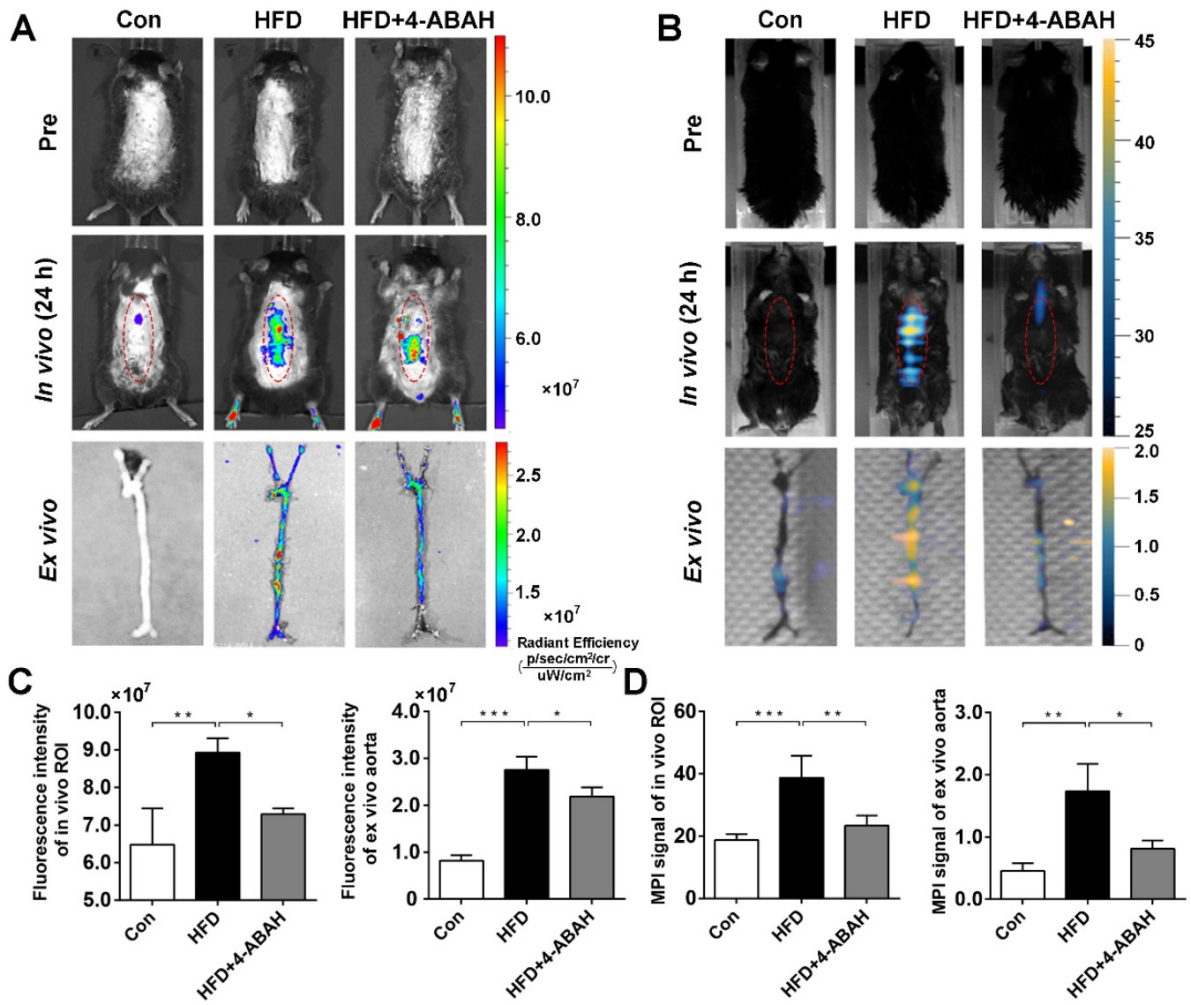
** FLI/MPI imaging of active MPO in aorta atheroma from mice in different groups after intravenous injection of 5HFeC NPs.** *In vivo* and *ex vivo* FLI of active MPO in aorta atheroma of ApoE^-/-^ mice before, and 24 h post, injection of 5HFeC NPs (A). *In vivo* and *ex vivo* MPI imaging of active MPO in aorta atheroma of ApoE^-/-^ mice before, and 24 h post, injection of 5HFeC NPs (B). Quantitative analysis of fluorescence intensity of *in vivo* ROI and *ex vivo* aorta (C). Quantitative analysis of MPI signal of *in vivo* ROI and *ex vivo* aorta (D). Red ellipse represents the ROI in mice. (n = 3 per group; *: P < 0.05; **: P < 0.005; ***: P < 0.001)

After intravenous injection of 5HFeC NPs, *in vivo* fluorescence images at 24 h post-injection (Figure 5A) revealed that fluorescence intensity was significantly higher in the HFD group than in the Con group (8.93 ± 0.22 × 10^7^ *vs.* 6.48 ± 0.56 × 10^7^, P < 0.005, respectively) and the HFD + 4-ABAH group (8.93 ± 0.22 × 10^7^ *vs.* 7.29 ± 0.09 × 10^7^, P < 0.05, respectively; Figure 5C). After extracting the carotid arteries and aorta, *ex vivo* fluorescence images of the vessels were acquired and analyzed (Figure 5A). *Ex vivo* fluorescence intensity in the aorta was significantly higher in the HFD group than in the Con group (2.75 ± 0.28 × 10^7^ *vs.* 0.82 ± 0.12 × 10^7^, P < 0.001, respectively; Figure 5C). 4-ABAH significantly reduced the fluorescence intensity of the aorta in the HFD + 4-ABAH group (2.75 ± 0.28 × 10^7^ *vs.* 2.19 ± 0.19 × 10^7^, P < 0.05, respectively). As revealed in Figure 5B and 5D, the MPI signal in the HFD group was significantly higher than that in the Con group (38.72 ± 6.99 *vs.* 18.77 ± 1.97, P < 0.001, respectively) and HFD + 4-ABAH group (38.72 ± 6.99 *vs.* 25.07 ± 3.72, P < 0.005, respectively). *Ex vivo* MPI signal in the aorta was significantly higher in the HFD group than in the Con group (1.74 ± 0.25 *vs.* 0.46 ± 0.07, P < 0.005, respectively) and HFD + 4-ABAH group (1.74 ± 0.25 *vs.* 0.81 ± 0.08, P < 0.05, respectively; Figure 5B and 5D).

### Histological analysis

**Figure 6:**
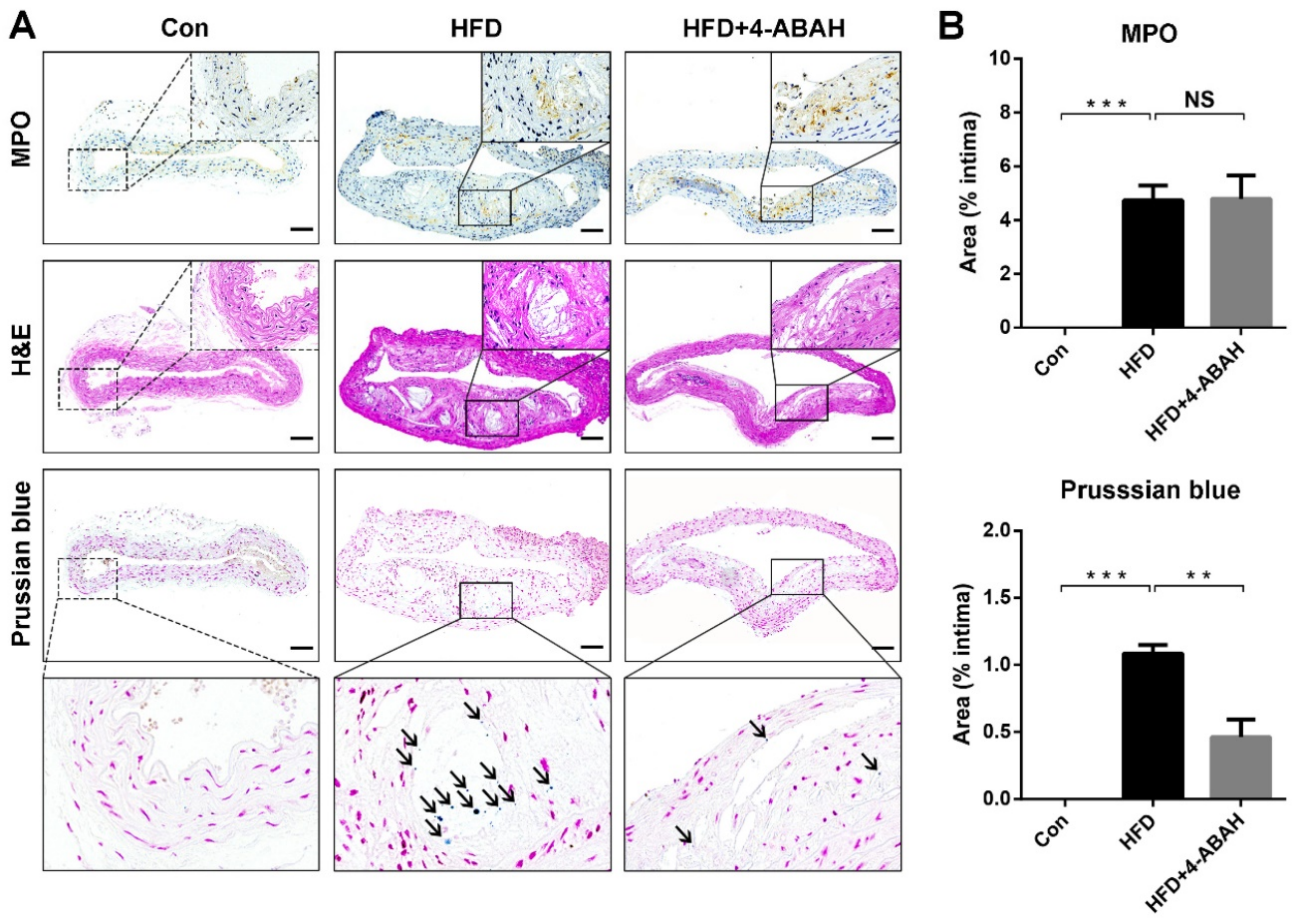
** MPO immunohistochemical, H&E, and Prussian blue staining of abdominal aorta tissues extracted from mice in different groups.** The black solid square in the MPO immunohistochemical staining image indicates the MPO-positive area in the atherosclerotic plaque from the HFD and HFD + 4-ABAH groups (A). The black solid square in the H&E staining image indicates the same area as the MPO immunohistochemical staining image and exhibits no evidence of intraplaque hemorrhage. The black solid square in the Prussian blue staining image indicates the same area as the black dashed square and shows the accumulation of 5HFeC NPs. The black arrow indicates the 5HFeC NPs (Prussian blue staining-positive area). The control (Con) group developed no atherosclerotic plaques in the intima, in which MPO immunohistochemical and Prussian blue staining showed almost no positive area. Quantification of MPO immunohistochemical and Prussian blue staining in the intima collected from the Con, HFD, and HFD + 4-ABAH groups (B). L, lumen; Scale bar, 100 μm. (n = 3 per group; NS: non-significant differences; **: P < 0.005; ***: P < 0.001).

Figure 6A shows the results of immunohistochemical staining of MPO, H&E and Prussian blue staining of atherosclerotic plaques from the abdominal aorta. Immunohistochemical staining revealed that MPO was expressed in the atherosclerotic plaques of the HFD and HFD + 4-ABAH groups, but not in the control group. The histology analysis showed that 5HFeC NPs were preferentially accumulated in the MPO-positive area of the HFD group but not in the HFD + 4-ABAH group. Further quantitative analysis (Figure 6B) indicated that the expression of MPO showed no difference between plaques in the HFD and HFD + 4-ABAH groups (4.74 ± 0.32% *vs.* 4.80 ± 0.50%, P > 0.05, respectively). 5HFeC NPs, stained by Prussian blue, accumulated more in the HFD group than in the HFD + 4-ABAH group (1.09 ± 0.04% *vs.* 0.46 ± 0.08%, P < 0.005, respectively). The Con group showed no expression of MPO or Fe accumulation in the intima.

To confirm the effect of MPO activity inhibition on the vulnerability of abdominal aorta plaques, the components of abdominal aorta plaques were further investigated in the HFD and HFD + 4-ABAH groups (Figure S8A and S8B). The plaques in the HFD + 4-ABAH group contained fewer macrophages (5.40 ± 1.28% *vs.* 12.11 ± 0.42%, P < 0.05, respectively) and smooth muscle cells (4.96 ± 1.24% *vs.* 9.46 ± 1.03%, P < 0.05, respectively) than did those in the HFD group. Neovessel (2.60 ± 0.60% *vs.* 3.10 ± 0.29%, P > 0.05, respectively), collagen (39.86 ± 3.67% *vs.* 41.59 ± 2.57%, P > 0.05, respectively), and microcalcification (1.58 ± 0.16% *vs.* 1.90 ± 0.20%, P > 0.05, respectively) showed no significant difference between plaques in the HFD + 4-ABAH and HFD groups. Moreover, MCP-1 was significantly lower in plaques of the HFD + 4-ABAH group than in those of the HFD group (5.14 ± 1.01% *vs.* 10.99 ± 0.85%, P < 0.05, respectively).

## Discussion

Globally, rupture of vulnerable plaques is the main cause of death in patients with atherosclerosis 1. The lack of an imaging technique capable of accurately identifying vulnerable atherosclerotic plaques is a major limitation in contemporary cardiovascular medicine 8. In this study, 5HFeC NPs were developed as novel imaging probes for detecting vulnerable plaques by targeting active MPO via FLI/MPI/CTA multimodality imaging.

Vulnerable plaques tend to have large lipid cores, abundant inflammatory cells, few smooth muscle cells, a thin fibrous cap, microcalcification, intraplaque hemorrhage, neovessels, and a necrotic core 32, 33. Molecular imaging tracers have been established to noninvasively visualize atherosclerotic plaques 8, 34. ^18^F-fluorodeoxyglucose is a commonly used radiotracer that can be taken up by activated inflammatory cells, leading to its use as a surrogate of vascular inflammation in atherosclerosis 4. Moreover, Qiao *et al.* 35 reported a novel osteopontin-targeted nanoprobe, which was applied in both MRI and FLI of macrophages in atherosclerosis *in vivo*. Jenkins *et al.* 36 used the PET radiotracer 18F-fluciclatide to target the α_v_β_3_-integrin receptor, which was upregulated in angiogenesis and inflammation under atherosclerosis conditions *in vivo*. Due to their low positive predictive values, these imaging tracers require further evidence to confirm their role in discriminating vulnerable from stable plaques 37. In our study, both FLI and MPI/CTA imaging demonstrated that 5HFeC NPs can target active MPO in atherosclerosis. Furthermore, the fluorescence intensity and MPI signal were primarily detected in the abdominal aorta rather than the aortic arch, indicating higher expression of active MPO in plaques in the abdominal aorta. Furthermore, histological analysis showed that atheroma in the abdominal aorta exhibited characteristics of vulnerability, including higher expression of macrophages, as well as more neovessels and microcalcification, and less collagen, than atheroma in the abdominal aorta. Moreover, MCP-1, a direct mediator of plaque instability that induces expression of metalloproteinases, cytokines, and adhesion molecules 38, was elevated in the atheroma of the abdominal aorta. These results suggest that 5HFeC NPs are useful for identifying vulnerable atherosclerotic plaques. Moreover, inhibition of MPO activity had no significant effect on the plaque size, but did reduce the fluorescence intensity and MPI signal of the 5HFeC NPs in the abdominal aorta. Histological analysis confirmed the reduced targeting by 5HFeC NPs of active MPO. Component analysis of plaques further demonstrated that inhibition of MPO activity stabilized the plaques in the abdominal aorta by reducing macrophages, smooth muscle cells, and expression of MCP-1. Thus, 5HFeC NPs with FLI/MPI/CTA can also be used to monitor MPO activity *in vivo*.

Molecular imaging probes should have low toxicity and should be biocompatible, stable, and specifically targeted for clinical application. Here, the hydrodynamic sizes of 5HFeC NPs were relatively stable after storage in PBS solution for one week, demonstrating good stability. Cell counting kit-8 assays demonstrated that 5HFeC NPs are not toxic to macrophages below a certain threshold (100 μg/mL). According to pathological examination of major organs extracted from MPO-implanted mice, no marked damage was detected in the liver, spleen, or other organs. *In vitro* oligomerization confirmed the specificity of 5HFeC NPs to MPO. Notably, 5-HT-based NPs were highly sensitive to MPO *in vivo*, with MPO-implanted thighs showing 0.84- and 1.45-fold higher fluorescence intensity and MPI signal than PBS-implanted thighs at 72 h in FLI and MPI, respectively. Injection of Fe_3_O_4_-Cy7 also resulted in an increase of fluorescence intensity and MPI signal in the MPO-implanted thigh. This may be due to the embedded Matrigel as exogenous implantation may activate acute inflammation and cause localized edema in the left thigh. The enhanced vascular permeability may then contribute to accumulation of a small amount of Fe_3_O_4_-Cy7 in the left thighs. Therefore, the relative fluorescence intensity and MPI signal were both lower than those in mice injected with 5HFeC NPs. Meanwhile, 72 h post-injection was determined to be the optimal time point for FLI imaging; however, 5-HT-based NPs clearly visualized active MPO via MPI imaging from 6 to 72 h post-injection.

Previous studies have also reported that Fe_3_O_4_ NPs typically accumulate in the liver and spleen 39. In this study, Prussian blue staining of the main organs confirmed the accumulation of 5HFeC NPs in the liver and spleen. However, the liver and spleen were removed during the MPI imaging process to reduce their effects on visualizing active MPO in the aorta plaque. However, *ex vivo* FLI revealed fluorescence signals in the kidney, lung, stomach, liver, and spleen, which may have been caused by the 5HFeC NP solution being mixed with a very small amount of Cy7-NHS after centrifugation. Meanwhile, Cy7-NHS accumulated in the kidney, lung, and stomach due to its small size. Despite the fact that the biggest challenge MPI faces is the poor spatial resolution of current MPI scanners, the 5HFeC NPs developed in our study showed a better resolution of 2.76 mm than did the other MPI tracers, such as Vivotrax (3.45 mm, Magnetic Insight Inc, USA) and Perimag (2.97 mm, Micromod Partikeltechnologie GmbH, Germany). Moreover, the MPI signal of 5HFeC NPs was higher than that of Vivotrax and Perimag at the same Fe concentration. Indeed, contrast agents have been combined with molecular imaging to visualize MPO activity in atherosclerotic plaques. For instance, Gd-*bis*-5-HT-diethylene triamine pentaacetic acid (Gd-*bis*-5-HT-DTPA) was used to noninvasively track MPO activity in atherosclerotic plaques in the thoracic aorta of a rabbit model *via* MRI 40. However, due to the low sensitivity of MRI, large doses of contrast agents are required to detect focal lesions, reducing their biological safety. Another study used ^111^In-*bis*-5HT-DTPA to image MPO-expressed atherosclerotic plaques in the murine aorta by single-photon emission computerized tomography (SPECT)/CT imaging 41. Despite the high sensitivity of SPECT, ionizing radiation creates challenges in nuclear imaging. Further, sulfonaphthoaminophenyl fluorescein (SNAPF), a fluorescent probe, was developed to detect MPO-expressing macrophages in frozen sections of human atherosclerotic carotid arteries 42. However, the excitation and emission wavelengths required limit its application for *in vivo* imaging.

Multimodality molecular imaging is a promising approach for non-invasively observing the atherosclerotic plaque composition, disease progression, and severity *in vivo* 8. Here, based on a multimodality probe, FLI and MPI/CTA were applied to visualize active MPO for vulnerable plaque identification. Although FLI has certain disadvantages, including autofluorescence perturbation and limited tissue penetration, its high sensitivity enables imaging of atherosclerotic plaques in superficial arteries, including the abdominal aorta and carotid artery 11. Furthermore, MPI can overcome the disadvantages of FLI due to its zero tissue depth signal attenuation, high sensitivity, and no ionizing radiation 12. CTA can assess the degree of luminal stenosis, image calcification, and provide anatomical information to achieve 3D imaging. The aortic diameter in the ApoE^-/-^ mouse imaged by MPI/CTA was approximately 1.5 mm, which is significantly smaller than that of the human coronary artery (3.5‒4 mm) and common carotid artery (6‒7.5 mm). Hence, the sizes of atherosclerotic plaques and vessels have limited effects on MPI/CTA imaging to detect human vulnerable plaques in patients with MI or stroke. Moreover, 5HFeC NPs in the atherosclerotic mice can be detected by MPI/CTA and FLI at 24 and 72 h post-injection. Long periods of circulation for MPI tracers have been reported previously. For instance, Zheng *et al.* used MPI to track SPIONs-labeled mesenchymal stem cells in lung tissues over 12 days 43. Another study monitored SPION-labeled neural progenitor cells in brain diseases *via* MPI imaging over 3 months 44. For the MPO-implanted mouse model in this study, metabolism of 5HFeC NPs in the MPO-implanted thigh can be monitored for 7 days. These results indicate the high sensitivity of FLI and MPI to detect active MPO. MPI has been utilized in various biomedical studies, including in cancer detection 39, stem cell imaging 43, exosome tracking 45, lung perfusion imaging 46, 47, and guidance of hyperthermia therapy 48, 49. However, ours is the first report on the use of multimodality FLI/MPI/CTA imaging for identifying vulnerable plaques.

To successfully predict the outcome of cardiovascular diseases, an ideal clinical imaging modality is required to image plaque composition and disease severity to differentiate stable from vulnerable plaques in the arteries. Several clinical approaches to atherosclerosis imaging have been well explored, such as coronary CTA, MRI, and nuclear imaging. However, no single modality offers a perfect solution to the imaging challenge, and each has its own disadvantages. Clinical coronary CTA can image the entire coronary artery tree with high temporal resolution; however, it is associated with radiation exposure and limited spatial resolution to adequately identify plaque characteristics 50. Although MRI has been used to detect neovessels 51, intraplaque hemorrhage 52, and plaque inflammation 5 in carotid arteries, plaque imaging of coronary arteries has proven challenging due to their small diameter, low spatial and temporal resolution and motion effects associated with the cardiac cycle and diaphragmatic movements 53. Meanwhile, PET combined with targeted radiotracers has been applied to assess vascular inflammation 4 and microcalcification 54 in clinical practice; however, it has been limited by its associated radiation exposure. In this study, MPI/CTA was shown to not only image the entire artery tree, but also sensitively identify vulnerable plaques located in the abdominal aorta through 3D whole-body imaging. Furthermore, it can be used to monitor inhibition of active MPO. This suggests that MPI/CTA is a promising imaging technique with great potential for detection of vulnerable atherosclerotic plaques, as well as monitoring MPO activity.

There are several limitations in our study. First, the liver and spleen must be removed when performing MPI imaging of active MPO in aortic plaques as histological analysis revealed that 5HFeC NPs were primarily localized in the liver and spleen, which was similar to the locations of other Fe_3_O_4_-based contrast agents 39. While signals from Fe-accumulating organs is an issue in mice due to proximity of the aorta to the liver (< 1 cm), this distance is significantly greater in humans and, thus, 6 T/m MPI imaging with 2.76 mm resolution can be expected to clearly detect atherosclerotic plaques in the aorta separate from any Fe accumulation in the liver. Moreover, the neck can be designated as the scanning area to detect vulnerable plaques in the carotid artery, the field of view for which is located away from the liver and spleen. Hence, the effects of these Fe-accumulating organs on MPI imaging in humans can be minimized. Moreover, 5HFeC NPs will be further optimized to accelerate their excretion from the liver and increase accumulation of MPO-targeting nanoparticles in atherosclerotic plaque, which can help enhance early detection of high-risk plaques and reduce the latency between nanoparticle administration and imaging. Second, MPI remains a preclinical imaging modality, which requires combination with CT to provide anatomical information. Recently, Graeser *et al.* reported a human-sized MPI brain scanner designed for monitoring cerebral perfusion in stroke patients in intensive care units 55. A hybrid MPI/MRI system was developed by Franke *et al.* 56 for non-invasive cardiovascular assessment as a preclinical investigation, which suggested that MPI combined with MRI can eliminate ionizing radiation in future studies. Thus, advanced MPI imaging for clinical use should be designed, which may open up a variety of medical applications in cardiovascular diseases and cancers. Finally, 5HFeC NPs with MPI/CTA imaging in the current study were tested only in mouse models. With the wide applications of SPIONs as MPI and MRI contrast agents, ferumoxytol 57, and ferucarbotran 17 have been approved for use in clinical practice. The SPIONs used in our study were tested in mice, but not in humans. Moreover, nanoparticles conjugated with Cy7-NHS, and 5-HT should be studied to confirm their biological safety in humans.

In conclusion, we successfully developed a specific nanoprobe (5HFeC NPs) that can be applied in FLI/MPI/CTA multimodality imaging of active MPO in atherosclerosis with high sensitivity. Furthermore, 5HFeC NPs may be useful for identifying vulnerable plaques and monitoring MPO activity.

## Methods

### Materials

The following materials were obtained from Sigma-Aldrich (St. Louis, MO, USA): 5-HT, *N*-(3-dimethylaminopropyl)-*N*ʹ-ethylcarbodiimide hydrochloride (EDC), and N-hydroxysuccinimide (NHS), lactoperoxidase (LPO). Fe_3_O_4_@PEG-COOH was purchased from Nanjing Nanoeast Biotech Co., Ltd. (Nanjing, China). Vivotrax and Perimag were purchased from Magnetic Insight Inc (Alameda, USA) and Micromod Partikeltechnologie GmbH (Rostock, Germany), respectively. Cy7-NHS was obtained from AAT Bioquest (Sunnyvale, CA, USA). RAW 264.7 cells (Otwo Biotech Inc., Shenzhen, China), Dulbecco's modified Eagle's medium (DMEM; Sigma-Aldrich), fetal bovine serum (FBS; Gibco, Grand Island, NY, USA), and cell counting kit-8 (CCK-8; Beyotime Institute of Biotechnology, Shanghai, China) were obtained for cell experiments. Myeloperoxidase (Abcam, Cambridge, UK), glucose oxidase (Dalian Meilun Biotechnology Co., Ltd., Dalian, China), Matrigel (Corning, Inc., Corning, NY, USA), 4-ABAH (Sigma-Aldrich), and ExiTron (TM) nano 12000 CT contrast agent (Miltenyi Biotec, Bergisch Gladbach, Germany) were purchased for animal experiments. Erythropoietin protein (EPO), rabbit anti-mouse MPO, rabbit anti-mouse CD68, rabbit anti-mouse anti-ɑ-smooth muscle actin (SMA), rabbit anti-mouse CD31, and rabbit anti-mouse MCP-1 antibodies were obtained from Abcam.

### Synthesis of 5-HT-Fe_3_O_4_-Cy7 and Fe_3_O_4_-Cy7

Fe_3_O_4_ NPs with active MPO targeting ability were synthesized as previously described 11. Fe_3_O_4_@PEG-COOH solution was dissolved in deionized water with pH 6 and activated with EDC and NHS. Subsequently, 5-HT was added to deionized water with pH 8.2 and dissolved in the mixed solution at a 3:1 molar ratio of 5-HT to carboxyl. The mixture was left to react overnight under continuous stirring at 20 °C and then centrifuged (5,580 × *g*, 30 min) to remove uncoupled 5-HT. The obtained 5-HT-Fe_3_O_4_ was dissolved in deionized water with pH 8.2. Cy7-NHS was added to react with the 5-HT-Fe_3_O_4_ solution in a 5:1 ratio of dye to carboxyl overnight at 20 °C with continuous stirring. The mixture was centrifuged (5,580 × *g*, 30 min) to remove uncoupled Cy7-NHS. Fe_3_O_4_-Cy7 was constructed by conjugating Cy7-NHS to the surface of Fe_3_O_4_@PEG-COOH. Cy7-NHS was added to react with the Fe_3_O_4_@PEG-COOH solution (pH = 8.2) in a 5:1 ratio of dye to carboxyl overnight at 20 °C with continuous stirring. The mixture was centrifuged (5,580 × *g*, 30 min) to remove uncoupled Cy7-NHS. All reactions were conducted in the dark by wrapping the centrifuge tube with aluminum foil.

### Characterization of 5-HT-Fe_3_O_4_-Cy7

5-HT-Fe_3_O_4_-Cy7 NPs were first prepared in deionized water. The iron oxide core size of 5HFeC NPs was measured by TEM (JEM-1200EX, JEOL, Tokyo, Japan). The average hydrodynamic particle size and zeta potential value of the nanoparticles were characterized using a Zetasizer Nano ZS90 (Malvern Instruments, Malvern, UK) at room temperature. Optical absorption spectra of the 5HFeC NPs and Fe_3_O_4_@PEG-COOH were obtained on an UV-Vis-NIR spectrophotometer (UV-3600 Plus, Shimadzu, Kumamoto, Japan). Both fluorescence emission spectra and excitation spectra of 5HFeC NPs and Cy7-NHS were recorded at room temperature with a fluorescence spectrofluorometer (F-7000, Hitachi, Tokyo, Japan). The PSF curves of 5HFeC NPs, Vivotrax, and Perimag were obtained with an MPI scanner (MOMENTUM, Magnetic Insight, Inc., Alameda, CA, USA), and the full width at half-maximum values were calculated with ImageJ software (1.8.0 version, National Institutes of Health, Bethesda, MD, USA). The fluorescence intensity and MPI signal of 5HFeC NPs were analyzed at various concentrations using IVIS spectrum (Caliper Life Sciences, PerkinElmer, Waltham, MA, USA) and an MPI scanner. Moreover, the MPI signals of Vivotrax and Perimag were obtained at the same Fe concentrations of 5HFeC NPs using the same MPI scanner. The hydrodynamic particle size of 5HFeC NPs was monitored daily for 7 days. The Powder XRD patterns of 5HFeC NPs were recorded on an X-ray Powder diffractometer (D8 ADVANCE, BRUCKER, Germany). The FT-IR analysis of Fe_3_O_4_ @PEG-COOH, 5-HT-Fe_3_O_4_, and 5HFeC NPs was performed using a Fourier infrared spectrometer (Nicolet IS10, Thermo Scientific, USA).

### Cell culture and cytotoxicity studies

RAW 264.7 cells were cultured in DMEM containing 10% FBS. Cytotoxicity was evaluated using CCK-8. Cells were seeded into 96-well plates (2000 cells/well) and incubated at 37 °C and 5% CO_2_ in an incubator (Heracell, Thermo Fisher Scientific, Waltham, MA, USA) for 24 h. The 5HFeC NPs were dissolved in DMEM containing 10% FBS at Fe concentrations of 0, 5, 10, 25, 50, 100, 150, 200 and 250 μg/mL. Next, the cells were treated with the nanoparticles and controlled medium (0 μg/mL Fe). After 24 h, the cells were washed with PBS three times and incubated in 100 μL DMEM containing 10 μL CCK-8 solution for 2 h. Optical density was measured at 450 nm and the values were expressed as a percentage of the control well values (Synergy HT Multi-Mode Microplate Reader, Biotek, Winooski, VT, USA).

### *In vitro* specific targeting ability

5HFeC NPs (1 mg/mL Fe concentration) in PBS (100 μL of total volume) with 30% H_2_O_2_ (10 μL) were incubated with MPO (8 μg, 1 mg/mL), EPO (5.2 μg, 1 mg/mL) or LPO (11.4 μg, 2 mg/mL) for 1 h at 37 °C, and with PBS without enzyme added as control. Subsequently, 5HFeC NPs from different groups were imaged using TEM.

### Animal experiments

All mice used in this study were purchased from Beijing Vital River Laboratory Animal Technology Co., Ltd. (Beijing, China). All animal studies and procedures were performed according to a protocol approved by the Chinese PLA General Hospital Animal Care and Use Committee in accordance with the National Institutes of Health Guideline on the Care and Use of Laboratory Animals.

### Animal model of MPO embedded in Matrigel implants

A 200-μL mixture of basement membrane Matrigel was slowly injected into the thighs of 8-week-old male C57BL/6J mice (n = 3) as previously described 58. Briefly, the mixture containing MPO (10 units) and glucose oxidase (5 units) was injected into the left thigh, whereas the mixture containing a similar volume of PBS was injected into the right thigh and served as a control. Matrigel was used to immobilize MPO and glucose oxidase, which supplies an environment of oxidative stress for oligomer formation or specific protein binding of 5HFeC NPs. At 1 h post-injection of Matrigel implants, the mice were intravenously administered 5HFeC NPs or Fe_3_O_4_-Cy7 (7.5 mg Fe per kg weight) for *in vivo* imaging.

### Animal model of atherosclerosis fed with HFD

Eight-week-old male ApoE^-/-^ mice (C57BL/6J background) were first fed a standard laboratory chow diet for one week. Next, 16 animals were randomly assigned to two groups: HFD group (n = 8) and HFD with MPO inhibitor (HFD + 4-ABAH) group (n = 8). Mice in the HFD group were fed a high-fat and high-cholesterol diet (containing 15% fat and 1.25% cholesterol by weight). Mice in the HFD + 4-ABAH group were administered the specific irreversible MPO inhibitor 4-ABAH (40 mg/kg) by intraperitoneal injection twice per day in addition to being fed the high-fat and high-cholesterol diet. Administration of 4-ABAH was performed for the duration of the experiment (40-42 weeks). Eight-week-old male C57BL/6J mice (n = 8) were fed the standard laboratory chow diet as a control group. After 40-42 weeks, the mice were intravenously administered 5HFeC NPs (7.5 mg Fe per kg weight) for imaging experiments. The mice were fasted for 24 h before the imaging experiments.

### Fluorescence imaging

To confirm the *in vivo* specificity of 5HFeC NPs and monitor their metabolism in MPO-implanted mice, FLI was performed by determining the IVIS spectrum after intravenous injection of 5HFeC NPs or Fe_3_O_4_-Cy7. Mice were first anesthetized with 1.5% isoflurane and FLI was conducted pre-injection as well as after 6, 12, 24, 48, 72, 96, 120, 144, and 168 h. The excitation and emission wavelengths were set to 724 and 788 nm, respectively. At 6 and 168 h post-injection, the mice were deeply anesthetized and euthanized to harvest the liver, spleen, stomach, lung, kidney, heart, intestine, left thigh, and right thigh for *ex vivo* imaging. *In vivo* FLI of atherosclerotic mice was performed before intravenous injection of 5HFeC NPs, as well as after 24 h and 72 h. At 72 h post-injection, the carotid arteries and aorta of mice were carefully excised and washed with PBS for *ex vivo* imaging. Quantitative analyses of *in vivo* and *ex vivo* imaging results were performed using Living Image Software (Caliper Life Sciences). MPO-implanted mice were analyzed by quantifying the relative fluorescence intensity (RFI) as follows: RFI = (L_AFI_ - R_AFI_)/R_AFI_, where L_AFI_ is the average fluorescence intensity within the ROI of the left thigh and R_AFI_ is the average fluorescence intensity within the ROI of the right thigh.

### MPI imaging

MPI imaging of the mice was performed using an MPI scanner (MOMENTUM, Magnetic Insight, Inc., Alameda, CA, USA) with a magnetic field gradient strength of 6 T/m. Mice were scanned along the z-axis of the scanner, with the excitation field (45kHz, 20mT peak amplitude) along the z-axis. MPO-implanted mice were anesthetized with a mixture of ketamine (100 mg/kg) and xylazine (10 mg/kg) before imaging. Subsequently, two-dimensional (2D) MPI imaging was performed at pre-injection, as well as after 6, 12, 24, 48, 72, 96, 120, 144, and 168 h. The scanner parameters were as follows: field of view (FOV): 6 × 4 cm; scan mode: isotropic; total time: 3 min. For 3D MPI imaging, images were captured with a FOV of 6 × 6 × 4 cm, scan mode: isotropic, number of projections = 35, total time: 30 min. MPO-implanted mice were analyzed by quantifying the relative MPI signal (RMS) as follows: RMS = (L_AMS_ - R_AMS_)/R_AMS_, where L_AMS_ is the average MPI signal within the ROI of the left thigh and R_AMS_ is the average MPI signal within the ROI of the right thigh. To observe the MPI images of atherosclerotic plaques, atherosclerotic mice were sacrificed at 24 h post-injection and the liver and spleen were removed for examination as these organs exhibit high uptake of nanoparticles. 2D MPI imaging was immediately performed using the following parameters: FOV: 6 × 10 cm; scan mode: isotropic; total time: 5 min. For 3D MPI imaging, the parameters were as follows: FOV: 6 × 6 × 10 cm, scan mode: isotropic, number of projections = 35, total time: 55 min. VivoQuant software (VivoQuant 4.0, Invicro, Boston, MA, USA) was used to analyze the MPI images.

### CT imaging

CT imaging was performed using a home-made micro-CT scanner. For MPO-implanted mice, the CT scan parameters were as follows: numbers of projections = 288; voltage: 90 kV; current: 88 μA, FOV: 100 mm; voxel size: 144 μm; scan time: 7 min. CT angiography was performed using a micro-CT (Quantum GX μCT System, PerkinElmer, Waltham, MA, USA) scanner. Atherosclerotic mice were intravenously injected with 100 µL ExiTron (TM) nano 12000 CT contrast agents 1 h prior to CT angiography. The CT scanner parameters were as follows: voltage: 90 kV; current: 88 μA, FOV: 45 mm; voxel size: 90 μm; scan mode: high-resolution; scan time: 14 min.

### MPI/CT co-registration

Fiducial markers (2 µL of 5HFeC NPs at a concentration of 1 mg Fe/mL) were sealed in a plastic tube and fixed on the animal bed, and used for 2D and 3D registration of the MPI images with CT images. 3D MPI/CT was rigidly co-registered by VivoQuant software.

### Histological analysis and immunohistochemistry

To assess the accumulation of 5HFeC NPs in the MPO-implanted area, the thighs and organs (heart, liver, spleen, lung, and kidney) of the MPO-implanted mice were extracted and embedded in 4% paraformaldehyde for histological sectioning. Prussian blue staining was performed to assess the accumulation of 5HFeC NPs in these tissues. Immunohistochemistry staining was performed to further confirm the expression of MPO in the thighs. Briefly, the thighs were embedded in optimal cutting temperature compound, frozen in liquid nitrogen, and stored at -20 °C until sectioning. Cryosections (5-µm-thick) were prepared, fixed in 4 °C acetone, and stained with anti-MPO antibody. To verify the formation of atherosclerotic plaques, the carotid arteries and aorta were isolated, embedded in 4% paraformaldehyde for 20 min, and stained with pre-warmed Oil Red O solution for 10 min. Bright-field images were acquired after washing with 60% isopropanol. To assess the atherosclerotic plaques, sections from the aortic arch and abdominal aorta were stained with H&E, Prussian blue, von Kossa, and picrosirius red stains. Immunohistochemical staining was performed to verify the expression of MPO, CD68, ɑ-SMA, CD31, and MCP-1 in the atherosclerotic plaques. Briefly, the vessel segments were embedded in OCT and snap frozen. Transverse cryosections were collected (5-µm-thick), fixed in 4 °C acetone, and stained with anti-MPO (1:200 dilution; catalog number: ab208670; Abcam, Cambridge, UK), anti-CD68 (1:200 dilution; catalog number: ab125212; Abcam, Cambridge, UK), anti-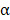-SMA (1:200 dilution; catalog number: ab5831; Abcam, Cambridge, UK), anti-CD31 (1:1000 dilution; catalog number: ab182981; Abcam, Cambridge, UK), and anti-MCP-1 (1:100 dilution; catalog number: ab8101; Abcam, Cambridge, UK) antibodies. Tissue slices were imaged using a slice scanning system (Zeiss Axio Scan. Z1, Carl Zeiss AG, Germany). For plaque assessment, the percentage of the various plaque components was quantified as the positive area in the intima for each specific parameter divided by the total intimal plaque area. For quantification of calcification, microcalcification was defined as the positive area with a diameter of 1-30 μm in the intima 59. ImageJ software (1.8.0 version) was used to perform quantitative analyses.

### Statistical analysis

Statistical analysis was performed using SPSS 23.0 statistical software (SPSS, Inc., Chicago, IL, USA). Data are presented as the means ± standard deviations. Comparisons between two groups were performed using Student's *t*-test. One-way analysis of variance was conducted among multi-groups followed by *post hoc* analysis using Dunnet's test. P < 0.05 was regarded as statistically significant.

## Supplementary Material

Supplementary figures.

Supplementary figures. Supplementary movie 1: 3D reconstruction of MPI/CT imaging of the 5H-Fe_3_O_4_-Cy7 nanoparticles in the MPO-implanted mouse.

Supplementary movie 2: 3D reconstruction of MPI/CTA imaging of the 5H-Fe_3_O_4_-Cy7 nanoparticles deposited in the aorta of the atherosclerotic mouse.

## Abbreviations

3D: three-dimensional
4-ABAH: 4-aminobenzoic acid hydrazide
5HFeC NPs: 5-HT- Fe_3_O_4_-Cy7 nanoparticles
5-HT: 5-hydroxytryptamine
CCK-8: cell counting kit-8
CTA: computed tomographic angiography
Cy7-NHS: cyanine 7 N-hydroxysuccinimide ester
DMEM: Dulbecco's modified Eagle's medium
EDC: *N*-(3-dimethylaminopropyl)-*N*'-ethylcarbodiimide hydrochloride
FBS: fetal bovine serum
FLI: fluorescence imaging
FOV: field of view
Gd-*bis*-5-HT-DTPA: Gd-*bis*-5-HT-diethylene triamine pentaacetic acid
H&E: hematoxylin and eosin
HFD: high-fat and high-cholesterol diet
MCP-1: monocyte chemoattractant protein-1
MI: myocardial infarction
MPI: magnetic particle imaging
MPO: myeloperoxidase
MRI: magnetic resonance imaging
NHS: N-hydroxysuccinimide
PBS: phosphate-buffered saline
PET: positron emission tomography
PSF: point spread function
RFI: relative fluorescence intensity
RMS: relative MPI signal
ROI: region of interest
SNAPF: sulfonaphthoaminophenyl fluorescein
SPECT: single-photon emission computerized tomography
SPIONs: superparamagnetic iron oxide nanoparticles
TEM: transmission electron microscopy
