# Highly sensitive magnetic particle imaging of vulnerable atherosclerotic plaque with active myeloperoxidase-targeted nanoparticles

**DOI:** 10.7150/thno.77886

**Published:** 2022-11-02

**Authors:** Wei Tong, Hui Hui, Wenting Shang, Yingqian Zhang, Feng Tian, Qiang Ma, Xin Yang, Jie Tian, Yundai Chen

**Affiliations:** 1 Medical School of Chinese PLA, Chinese PLA General Hospital, Beijing, 100853, China.; 2 Department of Cardiology, the Sixth Medical Centre, Chinese PLA General Hospital, Beijing, 100853, China.; 3 CAS Key Laboratory of Molecular Imaging, Institute of Automation, Chinese Academy of Sciences, Beijing, 100190, China.; 4 University of Chinese Academy of Sciences, Beijing, China.; 5 Beijing Advanced Innovation Center for Big Data-Based Precision Medicine, School of Medicine, Beihang University, Beijing, 100083, China.

The authors apologize that the original version of our paper unfortunately contained an incorrect representative image in Figure 4A, where the fluorescence image of an ApoE^-/-^ mouse acquired at 24 h post-injection was saved twice using different color scale bars of fluorescence intensity. We apologize that, during the image assembly, the fluorescence image acquired at 24 h post-injection was mistakenly chosen as the fluorescence image acquired before injection of 5HFeC NPs. The correct version of the Figure 4A is shown below. We confirm that this correction does not affect any original results and conclusions of the paper. The authors apologize for any inconvenience that the errors may have caused.

## Figures and Tables

**Figure 4A:**
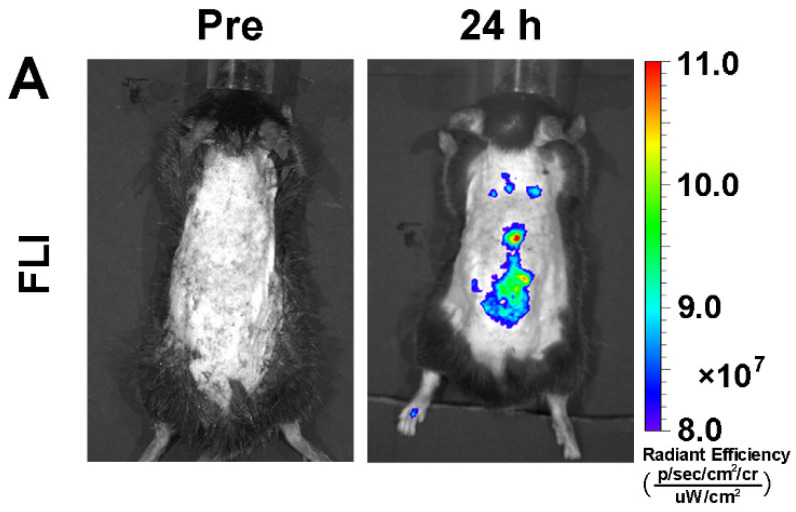
** (Corrected)** *In vivo* fluorescence image of active MPO in aorta atheroma of ApoE^-/-^ mice before, and 24 h post, injection of 5HFeC NPs.

