# Supplementary material for: Highly sensitive magnetic particle imaging of vulnerable atherosclerotic plaque with active myeloperoxidase-targeted nanoparticles
Source: Theranostics. 2021 Jan 1;11(2):506–21. doi: 10.7150/thno.49812 (PMC7738857; doi:10.7150/thno.49812)
Supplement: Supplementary file 1 — Supplementary figures. [file thnov11p0506s1.pdf]

Supplementary figures

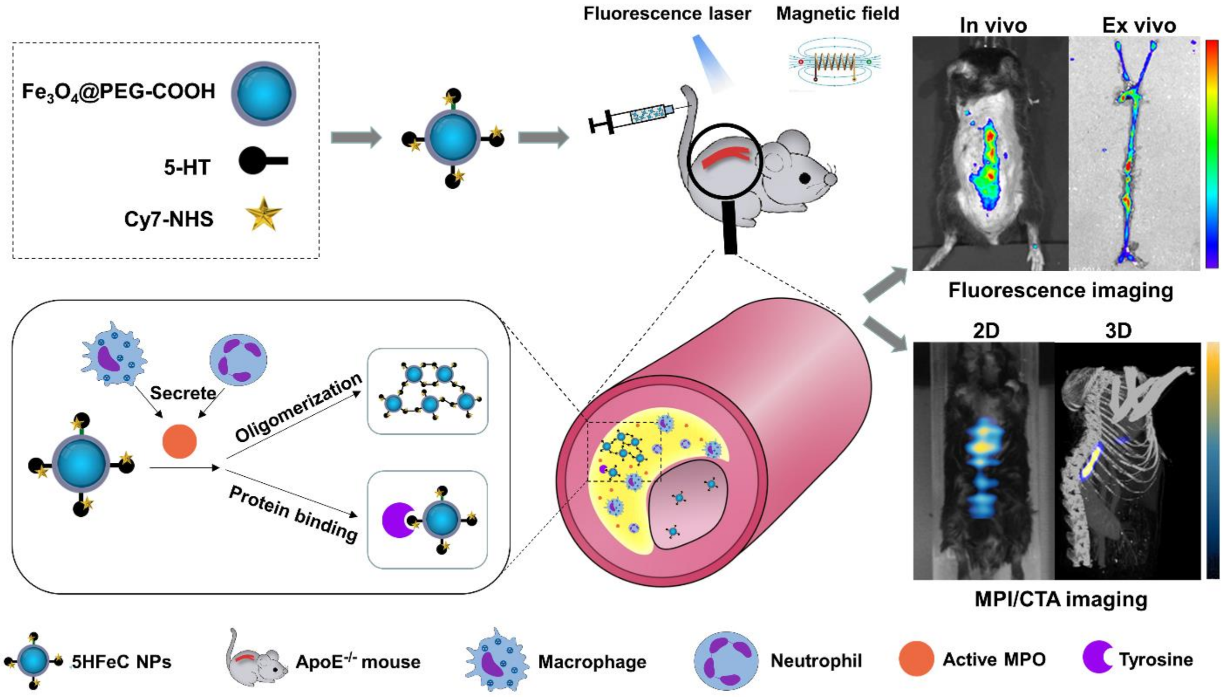

Figure S1. Schematic illustration of FLI/MPI/CTA imaging of active MPO to identify atherosclerotic vulnerable plaque. 5HFeC NPs were designed by conjugating 5-HT and Fe_3O_4@PEG-COOH with Cy7-NHS. Synthesized 5HFeC NPs were intravenously injected into ApoE$^{-/-}$ atherosclerotic mice and specifically targeted active MPO by oligomer formation or protein binding. Vulnerable plaque was identified *via* FLI/MPI/CTA imaging by active MPO targeting.

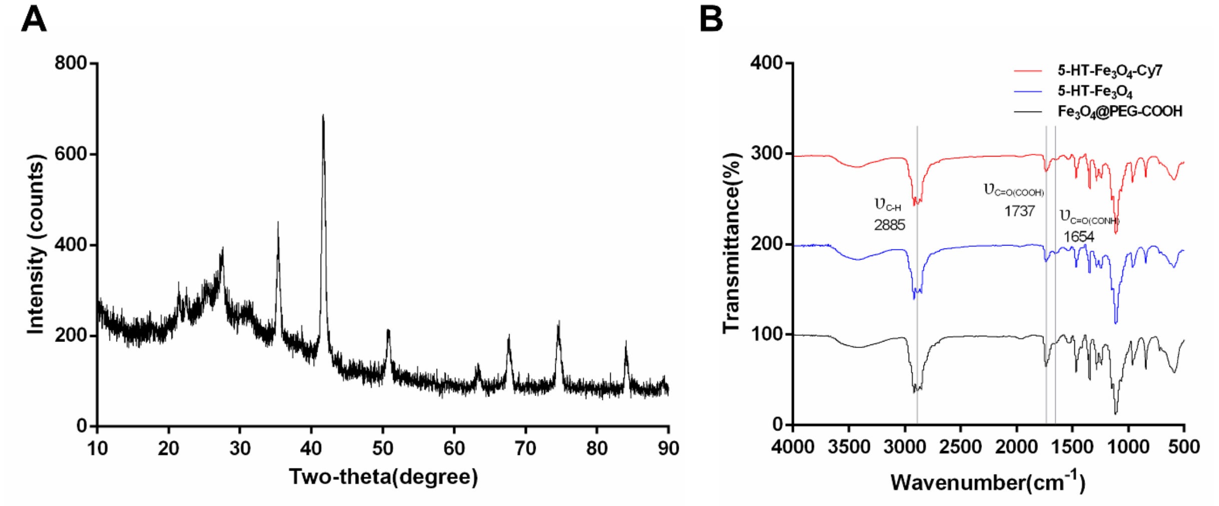

Figure S2. Powder XRD analysis of 5HFeC NPs (A) and fourier transform infrared spectra of Fe_3O_4 @PEG-COOH, 5-HT-Fe_3O_4 and 5HFeC NPs (B).

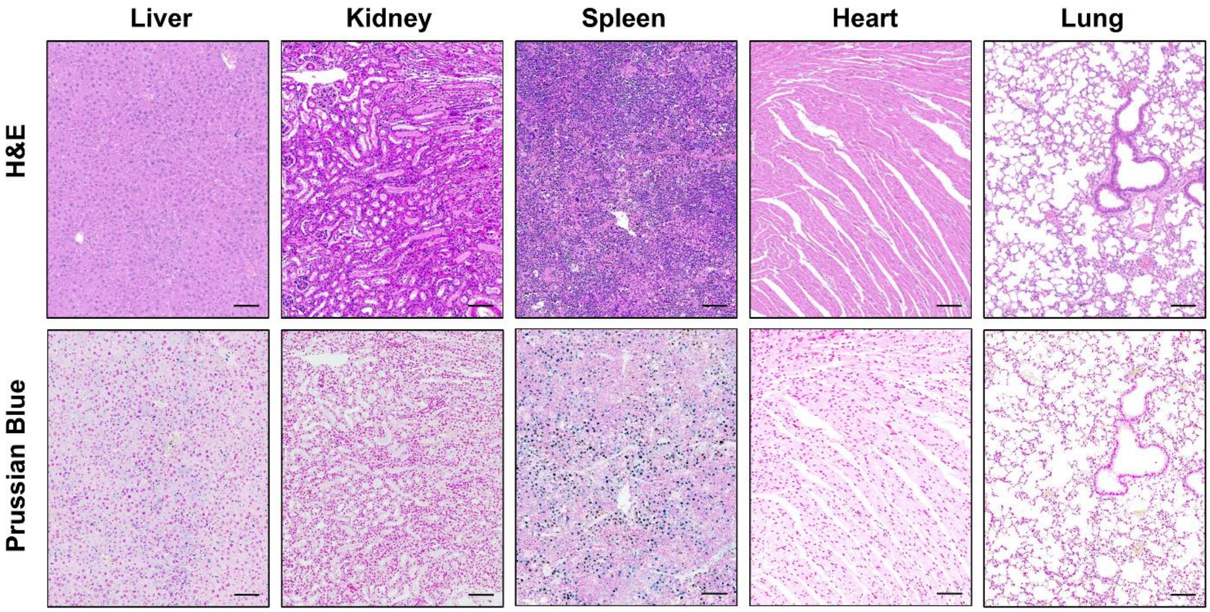

Figure S3. H&E and Prussian blue staining images of liver, kidney, spleen, heart and lung tissue sections of MPO-implanted mouse at 6 h after intravenous injection of 5HFeC NPs. Scale bar, 100 μm.

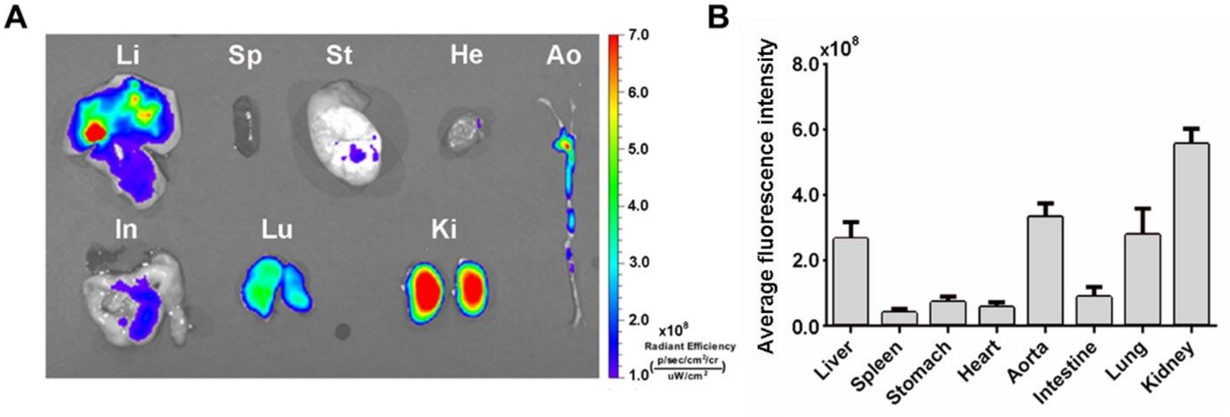

Figure S4. *Ex vivo* fluorescence imaging (A) and quantification analysis (B) of major organs at 24h after intravenous injection of 5HFeC NPs. Li, liver; Sp, spleen; St, stomach; He, heart; Ao, aorta; In, intestine; Lu, lung; Ki, kidney.

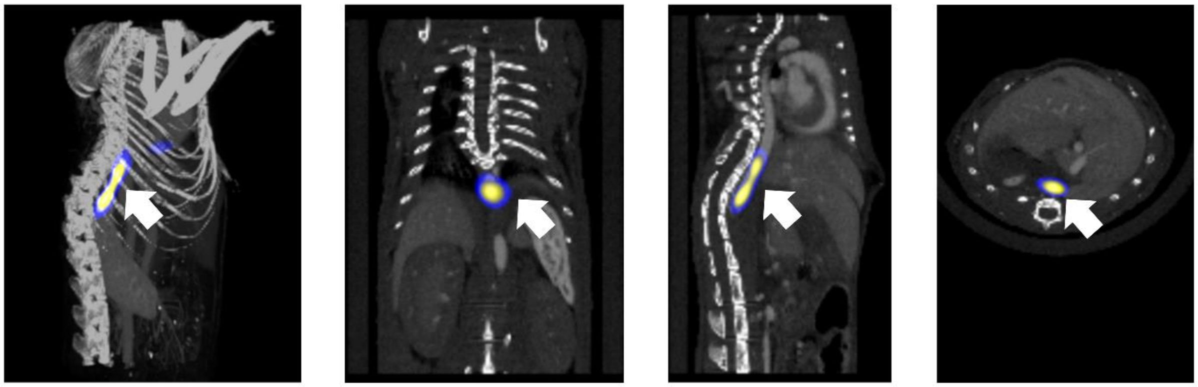

Figure S5. 3D MPI/CTA image (24 h post-injection) of atherosclerotic ApoE$^{-/-}$ mouse. Coronal, sagittal, and axial images through the aorta of the mice are also shown. White arrow indicates accumulation of 5HFeC NPs in the abdominal aorta.

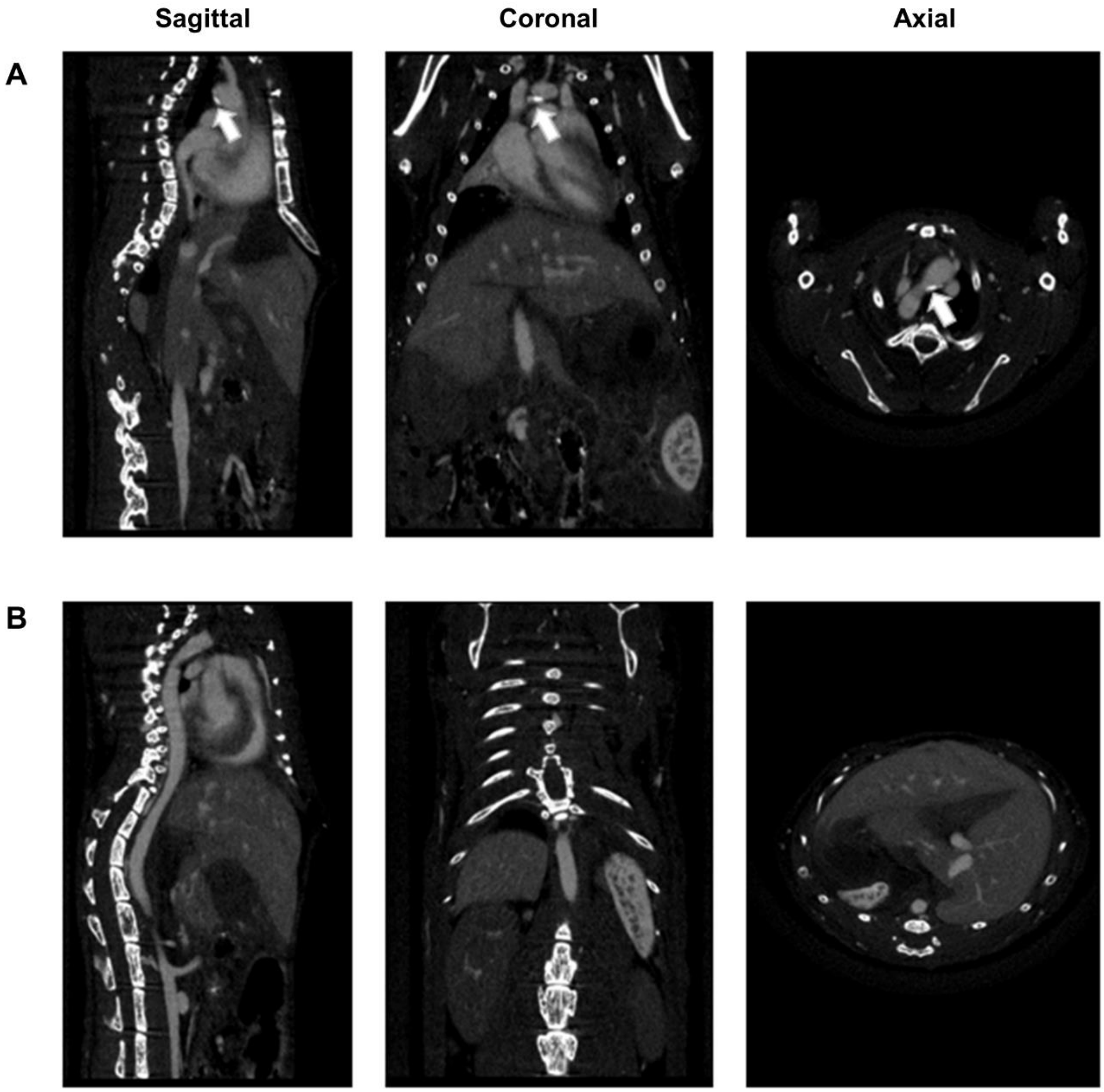

Figure S6. Calcification was detected in the aortic arch (A), rather than in the abdominal aorta (B) through CTA imaging of atherosclerotic mice. White arrow referred to calcification in the aorta.

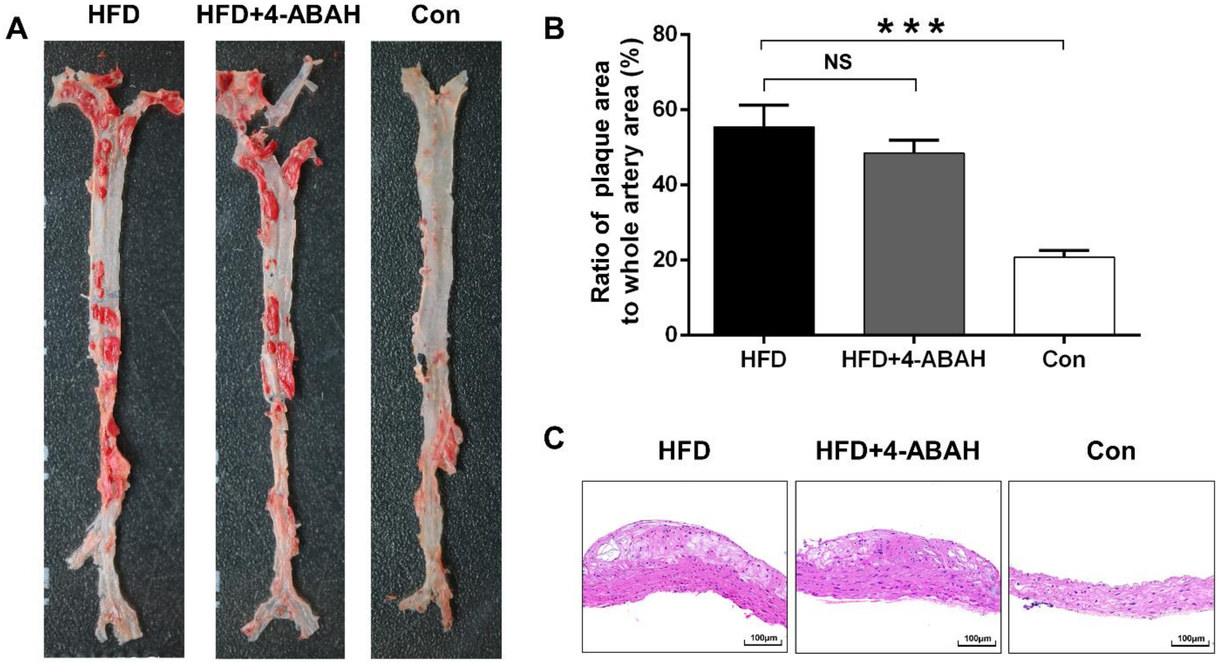

Figure S7. Representative Oil Red O staining images of aortas from different groups after 40-42 weeks feeding (A). Quantitation of mean Oil Red O stained plaque area (B). Representative images of H&E stained tissue sections from abdominal aorta of different groups (C). (n = 3 per group; NS: non-significant differences; ***: P < 0.001)

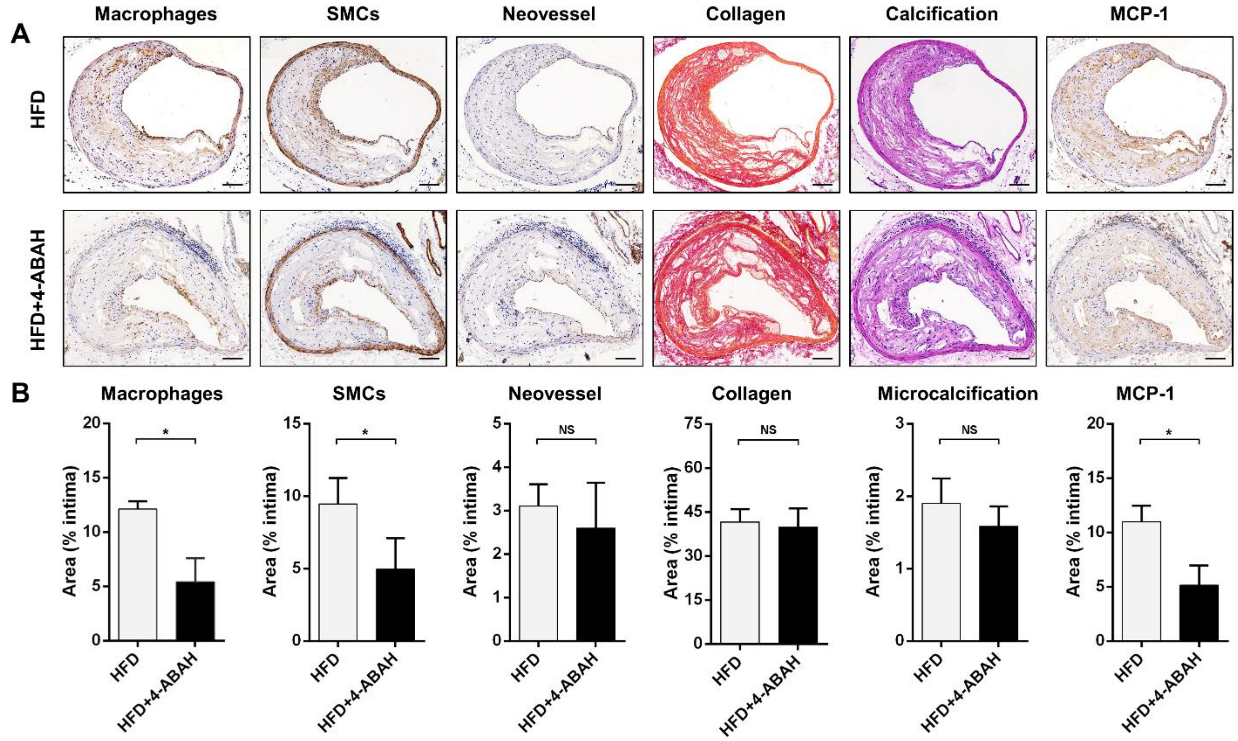

Figure S8. Cross-sections of plaque segments in the abdominal aorta collected from HFD and HFD + 4-ABAH group and stained for macrophages (CD68), SMCs (α-SMA), neovessel (CD31), calcification (von Kossa), collagen (picrosirius red), and MCP-1. SMCs, smooth muscle cells; MCP-1, monocyte chemoattractant protein-1-positive cells (A). Quantification of macrophages, SMCs, neovessel, microcalcification, collagen, and MCP-1 in the intimal area of plaque segments collected from HFD and HFD + 4-ABAH group (B). Scale bar in (A), 100 μm. (n = 3 per group; NS: non-significant differences; *: $P < 0.05$).
